# Hexaaqua­hexa­kis(μ_2_-3,5-diamino-4*H*-1,2,4-triazole)trinickel(II) tris­(hexa­fluoridosilicate) icosa­hydrate

**DOI:** 10.1107/S1600536808013329

**Published:** 2008-05-10

**Authors:** Li-Ping Wu, Shu-Ming Zhao, Guo-Fang Zhang, Seik Weng Ng

**Affiliations:** a Key Laboratory of Applied Surface and Colloid Chemistry, Shaanxi Normal University, Ministry of Education, Xi’an 710062, People’s Republic of China; b Department of Chemistry, University of Malaya, 50603 Kuala Lumpur, Malaysia

## Abstract

The trinuclear cation of the title compound, [Ni_3_(C_2_H_5_N_5_)_6_(H_2_O)_6_][SiF_6_]_3_·20H_2_O, has the six 3,5-diamino-1,2,4-triazole ligands each bridging two metal atoms; the metal atom in the middle, which lies on a special position (of 32 site symmetry), is connected to six N atoms in an octa­hedral geometry. The other metal atom, which lies on a special position (of 3 site symmetry), is connected to three N atoms and three O atoms. One hexa­fluroridosilicate anion lies on a site of 3 symmetry and the other lies on a site of 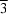 symmetry. The hexa­cation, dianions and uncoordinated water mol­ecules inter­act through hydrogen bonds to form a three-dimensional network. One uncoordinated water molecule is disordered, with site occupancy 0.3.

## Related literature

For the structure of the title hexa­cation as the hydrated sulfate salt, see: Zhang *et al.* (2007 ▶).
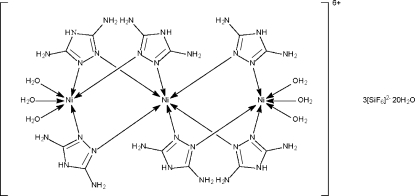


## Experimental

### 

#### Crystal data

[Ni_3_(C_2_H_5_N_5_)_6_(H_2_O)_6_][SiF_6_]_3_·20H_2_O
*M* *_r_* = 1665.48
Trigonal, 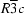

*a* = 13.024 (1) Å
*c* = 64.462 (5) Å
*V* = 9469.8 (9) Å^3^
*Z* = 6
Mo *K*α radiation
μ = 1.09 mm^−1^
*T* = 293 (2) K
0.49 × 0.46 × 0.44 mm

#### Data collection

Bruker APEX area-detector diffractometer
Absorption correction: multi-scan (*SADABS*; Sheldrick, 1996 ▶) *T*_min_ = 0.560, *T*_max_ = 0.646
25347 measured reflections
2420 independent reflections
2093 reflections with *I* > 2σ(*I*)
*R*_int_ = 0.021

#### Refinement

*R*[*F* ^2^ > 2σ(*F* ^2^)] = 0.033
*wR*(*F* ^2^) = 0.106
*S* = 1.06
2420 reflections
185 parameters
23 restraints
H atoms treated by a mixture of independent and constrained refinement
Δρ_max_ = 0.40 e Å^−3^
Δρ_min_ = −0.42 e Å^−3^

### 

Data collection: *SMART* (Bruker, 2004 ▶); cell refinement: *SAINT* (Bruker, 2004 ▶); data reduction: *SAINT*; program(s) used to solve structure: *SHELXS97* (Sheldrick, 2008 ▶); program(s) used to refine structure: *SHELXL97* (Sheldrick, 2008 ▶); molecular graphics: *X-SEED* (Barbour, 2001 ▶); software used to prepare material for publication: *publCIF* (Westrip, 2008 ▶).

## Supplementary Material

Crystal structure: contains datablocks I, global. DOI: 10.1107/S1600536808013329/si2084sup1.cif

Structure factors: contains datablocks I. DOI: 10.1107/S1600536808013329/si2084Isup2.hkl

Additional supplementary materials:  crystallographic information; 3D view; checkCIF report

## Figures and Tables

**Table 1:** Selected bond lengths (Å)

Ni1—N1	2.062 (2)
Ni1—O1	2.104 (2)
Ni2—N2	2.111 (2)

**Fig. 1.**
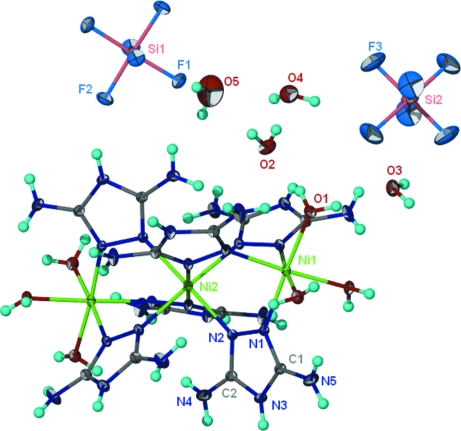
Thermal ellipsoid plot of the title compound. Displacement ellipsoids are drawn at the 25% probability level.

## supplementary crystallographic information

### Comment

A recent study reported the nickel sulfate complex of 3,5-diamino-1,2,4-triazole. The cation is a centrosymmetric trinuclear hexacation [Ni_3_(C_2_H_5_N_5_)_6_(H_2_O)_6_]^6+^, whose charge is balanced by the sulfate anions. The *N*-heterocyclic ligands each bridge two nickel atoms (Zhang *et al.*, 2007). The corresponding synthesis with nickel hexafluoridosilicate in place of nickel sulfate gave the analogous cluster cation (Scheme I, Fig. 1). The cation and anions interact through the coordinated and free water molecules to give rise to a three-dimensional, hydrogen bonded network.

### Experimental

Single crystal of the compound were grown by diffusing 3,5-diamino-1,2,4-triazole (0.020 g, 0.2 mmol) dissolved in methanol (5 ml) into nickle(II) hexafluorosilicate (0.027 g, 0.1 mmol) dissolved in water (5 ml).

### Refinement

As one of the five water molecules (O5) lies on a special position of *2* site symmetry, the occupancy would be 0.5. However, the refinement of this atom at the default occupancy lead to a large temperature factor. The refinement of the occupancy factor led to a value of about 0.3; this atom was then allowed to refine off the symmetry element. As the occupancy was nearly 0.3, the occupancy was arbitrarily fixed as 0.3333 so that the formula unit has 20 lattice water molecules.

The N– and O–bound H atoms (other than those of the diordered water molecule) were found in difference maps and were refined with distance restraints of O–H = N–H = O.85±0.01 Å; for the water molecules, an additional H···H = 1.39±0.01 Å restraint was imposed. The *U*_iso_(H) values were tied to those of the parent atoms by a factor of 1.5. The H atoms of the disordered water molecule were placed in chemically sensible positions but were not refined.

### Crystal data

**Table:**

[Ni_3_(C_2_H_5_N_5_)_6_(H_2_O)_6_][SiF_6_]_3_·20H_2_O	*Z* = 6
*M**_r_* = 1665.48	*F*_000_ = 5160
Trigonal, *R*3*c*	*D*_x_ = 1.752 Mg m^−^^3^
Hall symbol: -R 3 2"c	Mo *K*α radiation λ = 0.71073 Å
*a* = 13.024 (1) Å	Cell parameters from 5839 reflections
*b* = 13.024 Å	θ = 3.1–28.2º
*c* = 64.462 (5) Å	µ = 1.09 mm^−^^1^
α = 90º	*T* = 293 (2) K
β = 90º	Block, blue
γ = 120º	0.49 × 0.46 × 0.44 mm
*V* = 9469.8 (9) Å^3^	

### Data collection

**Table:**

Bruker APEX area-detector diffractometer	2420 independent reflections
Radiation source: fine-focus sealed tube	2093 reflections with *I* > 2σ(*I*)
Monochromator: graphite	*R*_int_ = 0.021
*T* = 293(2) K	θ_max_ = 27.5º
φ and ω scans	θ_min_ = 3.1º
Absorption correction: multi-scan(SADABS; Sheldrick, 1996)	*h* = −16→16
*T*_min_ = 0.560, *T*_max_ = 0.646	*k* = −16→16
25347 measured reflections	*l* = −79→83

### Refinement

**Table:**

Refinement on *F*^2^	Secondary atom site location: difference Fourier map
Least-squares matrix: full	Hydrogen site location: inferred from neighbouring sites
*R*[*F*^2^ > 2σ(*F*^2^)] = 0.033	H atoms treated by a mixture of independent and constrained refinement
*wR*(*F*^2^) = 0.106	*w* = 1/[σ^2^(*F*_o_^2^) + (0.0612*P*)^2^ + 17.0807*P*] where *P* = (*F*_o_^2^ + 2*F*_c_^2^)/3
*S* = 1.06	(Δ/σ)_max_ = 0.001
2420 reflections	Δρ_max_ = 0.40 e Å^−^^3^
185 parameters	Δρ_min_ = −0.42 e Å^−^^3^
23 restraints	Extinction correction: none
Primary atom site location: structure-invariant direct methods	

### Fractional atomic coordinates and isotropic or equivalent isotropic displacement parameters (Å^2^)

**Table:**

	*x*	*y*	*z*	*U*_iso_*/*U*_eq_	Occ. (<1)
Ni1	0.6667	0.3333	0.140787 (6)	0.02689 (14)	
Ni2	0.6667	0.3333	0.0833	0.02377 (16)	
Si1	1.0000	0.0000	0.083802 (16)	0.0331 (2)	
Si2	0.3333	−0.3333	0.1667	0.0552 (5)	
F1	0.89685 (14)	0.00226 (15)	0.09960 (2)	0.0623 (4)	
F2	1.00168 (16)	0.10635 (14)	0.06943 (3)	0.0648 (4)	
F3	0.3983 (3)	−0.3868 (3)	0.15169 (5)	0.1399 (12)	
O1	0.72286 (15)	0.24063 (14)	0.16010 (2)	0.0398 (3)	
H11	0.723 (3)	0.1843 (18)	0.1533 (3)	0.060*	
H12	0.699 (3)	0.217 (2)	0.1724 (2)	0.060*	
O2	0.7536 (2)	0.0854 (2)	0.13625 (4)	0.0660 (5)	
H21	0.713 (2)	0.0108 (10)	0.1358 (6)	0.099*	
H22	0.8267 (10)	0.108 (3)	0.1361 (7)	0.099*	
O3	0.65459 (19)	0.15616 (17)	0.19955 (3)	0.0599 (5)	
H31	0.5874 (17)	0.094 (2)	0.1978 (5)	0.090*	
H32	0.650 (3)	0.197 (2)	0.2092 (4)	0.090*	
O4	0.6041 (2)	−0.1661 (3)	0.12859 (4)	0.0796 (6)	
H41	0.576 (3)	−0.184 (4)	0.1164 (3)	0.119*	
H42	0.551 (3)	−0.182 (4)	0.1374 (4)	0.119*	
O5	0.664 (3)	−0.1549 (17)	0.0893 (3)	0.185 (7)	0.3333
H51	0.7382	−0.1072	0.0905	0.278*	0.3333
H52	0.6338	−0.1239	0.0816	0.278*	0.3333
N1	0.61588 (14)	0.43372 (14)	0.12352 (2)	0.0290 (3)	
N2	0.59282 (14)	0.41343 (14)	0.10195 (2)	0.0293 (3)	
N3	0.52872 (17)	0.53100 (17)	0.11289 (3)	0.0381 (4)	
H3	0.500 (2)	0.576 (2)	0.1117 (4)	0.057*	
N4	0.5100 (2)	0.4865 (2)	0.07683 (3)	0.0508 (5)	
H4A	0.514 (3)	0.449 (3)	0.0663 (4)	0.076*	
H4B	0.468 (2)	0.517 (3)	0.0744 (6)	0.076*	
N5	0.5810 (2)	0.5478 (2)	0.14862 (3)	0.0556 (6)	
H5A	0.625 (3)	0.540 (3)	0.1574 (4)	0.083*	
H5B	0.535 (3)	0.575 (3)	0.1509 (5)	0.083*	
C1	0.57611 (18)	0.50413 (18)	0.12943 (3)	0.0343 (4)	
C2	0.54160 (17)	0.47436 (17)	0.09630 (3)	0.0336 (4)	

### Atomic displacement parameters (Å^2^)

**Table:**

	*U*^11^	*U*^22^	*U*^33^	*U*^12^	*U*^13^	*U*^23^
Ni1	0.02997 (17)	0.02997 (17)	0.0207 (2)	0.01499 (9)	0.000	0.000
Ni2	0.0253 (2)	0.0253 (2)	0.0207 (3)	0.01265 (10)	0.000	0.000
Si1	0.0300 (3)	0.0300 (3)	0.0394 (5)	0.01500 (15)	0.000	0.000
Si2	0.0333 (5)	0.0333 (5)	0.0992 (15)	0.0166 (2)	0.000	0.000
F1	0.0555 (9)	0.0683 (10)	0.0701 (10)	0.0363 (8)	0.0170 (7)	0.0016 (8)
F2	0.0710 (10)	0.0574 (9)	0.0741 (10)	0.0382 (8)	−0.0004 (8)	0.0199 (8)
F3	0.146 (3)	0.176 (3)	0.163 (3)	0.129 (3)	0.0160 (19)	−0.025 (2)
O1	0.0497 (8)	0.0454 (8)	0.0292 (7)	0.0274 (7)	−0.0020 (6)	0.0041 (6)
O2	0.0624 (12)	0.0722 (13)	0.0750 (13)	0.0424 (11)	−0.0019 (10)	−0.0133 (11)
O3	0.0764 (13)	0.0541 (11)	0.0436 (9)	0.0283 (10)	0.0132 (9)	−0.0001 (8)
O4	0.0709 (15)	0.0812 (15)	0.0871 (16)	0.0383 (13)	−0.0030 (12)	−0.0025 (14)
O5	0.119 (7)	0.216 (10)	0.178 (13)	0.051 (7)	−0.015 (9)	−0.010 (8)
N1	0.0349 (8)	0.0329 (8)	0.0226 (7)	0.0194 (6)	0.0006 (6)	−0.0010 (6)
N2	0.0352 (8)	0.0355 (8)	0.0219 (7)	0.0213 (7)	−0.0009 (6)	−0.0005 (6)
N3	0.0476 (10)	0.0440 (10)	0.0366 (9)	0.0334 (8)	−0.0010 (7)	−0.0015 (7)
N4	0.0743 (15)	0.0720 (14)	0.0343 (10)	0.0578 (13)	−0.0109 (9)	−0.0043 (9)
N5	0.0821 (16)	0.0803 (15)	0.0348 (10)	0.0635 (14)	−0.0071 (10)	−0.0157 (10)
C1	0.0390 (10)	0.0367 (10)	0.0319 (9)	0.0224 (9)	0.0008 (8)	−0.0023 (8)
C2	0.0377 (10)	0.0374 (10)	0.0312 (9)	0.0229 (9)	−0.0009 (7)	0.0006 (7)

### Geometric parameters (Å, °)

**Table:**

Ni1—N1	2.062 (2)	Si2—F3	1.649 (3)
Ni1—N1^i^	2.062 (2)	O1—H11	0.86 (3)
Ni1—N1^ii^	2.062 (2)	O1—H12	0.85 (3)
Ni1—O1	2.104 (2)	O2—H21	0.84 (3)
Ni1—O1^i^	2.104 (2)	O2—H22	0.84 (3)
Ni1—O1^ii^	2.104 (2)	O3—H31	0.85 (3)
Ni2—N2^iii^	2.111 (2)	O3—H32	0.84 (3)
Ni2—N2^iv^	2.111 (2)	O4—H41	0.85 (3)
Ni2—N2^v^	2.111 (2)	O4—H42	0.84 (3)
Ni2—N2^ii^	2.111 (2)	O5—H51	0.85
Ni2—N2^i^	2.111 (2)	O5—H52	0.85
Ni2—N2	2.111 (2)	N1—C1	1.315 (2)
Si1—F2	1.6574 (15)	N1—N2	1.419 (2)
Si1—F2^vi^	1.6574 (15)	N2—C2	1.318 (3)
Si1—F2^vii^	1.6574 (15)	N3—C2	1.356 (3)
Si1—F1^vii^	1.6976 (15)	N3—C1	1.362 (3)
Si1—F1^vi^	1.6976 (15)	N3—H3	0.85 (1)
Si1—F1	1.6976 (15)	N4—C2	1.354 (3)
Si2—F3^viii^	1.649 (3)	N4—H4A	0.85 (3)
Si2—F3^ix^	1.649 (3)	N4—H4B	0.84 (3)
Si2—F3^x^	1.649 (3)	N5—C1	1.350 (3)
Si2—F3^xi^	1.649 (3)	N5—H5A	0.84 (3)
Si2—F3^xii^	1.649 (3)	N5—H5B	0.85 (3)
			
N1^ii^—Ni1—N1^i^	93.61 (6)	F1^vi^—Si1—F1	87.74 (9)
N1^ii^—Ni1—N1	93.61 (6)	F3^viii^—Si2—F3^ix^	89.21 (17)
N1^i^—Ni1—N1	93.61 (6)	F3^viii^—Si2—F3^x^	89.21 (17)
N1^ii^—Ni1—O1	87.31 (6)	F3^ix^—Si2—F3^x^	89.21 (17)
N1^i^—Ni1—O1	90.45 (6)	F3^viii^—Si2—F3^xi^	90.79 (17)
N1—Ni1—O1	175.77 (6)	F3^ix^—Si2—F3^xi^	90.79 (17)
N1^ii^—Ni1—O1^i^	90.45 (6)	F3^x^—Si2—F3^xi^	179.995 (1)
N1^i^—Ni1—O1^i^	175.77 (6)	F3^viii^—Si2—F3^xii^	90.79 (17)
N1—Ni1—O1^i^	87.31 (6)	F3^ix^—Si2—F3^xii^	179.995 (1)
O1—Ni1—O1^i^	88.56 (6)	F3^x^—Si2—F3^xii^	90.79 (17)
N1^ii^—Ni1—O1^ii^	175.78 (6)	F3^xi^—Si2—F3^xii^	89.21 (17)
N1^i^—Ni1—O1^ii^	87.31 (6)	F3^viii^—Si2—F3	179.997 (1)
N1—Ni1—O1^ii^	90.45 (6)	F3^ix^—Si2—F3	90.79 (17)
O1—Ni1—O1^ii^	88.56 (6)	F3^x^—Si2—F3	90.79 (17)
O1^i^—Ni1—O1^ii^	88.56 (6)	F3^xi^—Si2—F3	89.21 (17)
N2^iii^—Ni2—N2^iv^	90.87 (6)	F3^xii^—Si2—F3	89.21 (17)
N2^iii^—Ni2—N2^v^	90.87 (6)	Ni1—O1—H11	110 (2)
N2^iv^—Ni2—N2^v^	90.87 (6)	Ni1—O1—H12	126 (2)
N2^iii^—Ni2—N2^ii^	87.57 (8)	H11—O1—H12	109 (2)
N2^iv^—Ni2—N2^ii^	177.79 (8)	H21—O2—H22	111 (2)
N2^v^—Ni2—N2^ii^	90.72 (8)	H31—O3—H32	110 (2)
N2^iii^—Ni2—N2^i^	90.72 (8)	H41—O4—H42	111 (2)
N2^iv^—Ni2—N2^i^	87.57 (8)	H51—O5—H52	109.4
N2^v^—Ni2—N2^i^	177.79 (8)	C1—N1—N2	107.04 (15)
N2^ii^—Ni2—N2^i^	90.88 (6)	C1—N1—Ni1	130.48 (12)
N2^iii^—Ni2—N2	177.79 (8)	N2—N1—Ni1	121.05 (11)
N2^iv^—Ni2—N2	90.72 (8)	C2—N2—N1	106.38 (15)
N2^v^—Ni2—N2	87.57 (8)	C2—N2—Ni2	129.31 (13)
N2^ii^—Ni2—N2	90.88 (6)	N1—N2—Ni2	122.87 (12)
N2^i^—Ni2—N2	90.88 (6)	C2—N3—C1	106.38 (16)
F2—Si1—F2^vi^	91.80 (9)	C2—N3—H3	122 (2)
F2—Si1—F2^vii^	91.80 (9)	C1—N3—H3	132 (2)
F2^vi^—Si1—F2^vii^	91.80 (9)	C2—N4—H4A	125 (2)
F2—Si1—F1^vii^	90.36 (8)	C2—N4—H4B	122 (3)
F2^vi^—Si1—F1^vii^	177.12 (10)	H4A—N4—H4B	111 (3)
F2^vii^—Si1—F1^vii^	90.03 (8)	C1—N5—H5A	117 (2)
F2—Si1—F1^vi^	177.12 (10)	C1—N5—H5B	116 (2)
F2^vi^—Si1—F1^vi^	90.03 (8)	H5A—N5—H5B	126 (3)
F2^vii^—Si1—F1^vi^	90.36 (8)	N1—C1—N5	127.37 (19)
F1^vii^—Si1—F1^vi^	87.74 (9)	N1—C1—N3	109.86 (16)
F2—Si1—F1	90.03 (8)	N5—C1—N3	122.76 (18)
F2^vi^—Si1—F1	90.36 (8)	N2—C2—N4	126.92 (19)
F2^vii^—Si1—F1	177.12 (10)	N2—C2—N3	110.33 (17)
F1^vii^—Si1—F1	87.74 (9)	N4—C2—N3	122.67 (19)
			
N1^ii^—Ni1—N1—C1	−135.1 (2)	N2^iv^—Ni2—N2—N1	146.76 (16)
N1^i^—Ni1—N1—C1	131.1 (2)	N2^v^—Ni2—N2—N1	−122.40 (15)
O1^i^—Ni1—N1—C1	−44.81 (19)	N2^ii^—Ni2—N2—N1	−31.71 (12)
O1^ii^—Ni1—N1—C1	43.72 (19)	N2^i^—Ni2—N2—N1	59.18 (10)
N1^ii^—Ni1—N1—N2	60.39 (10)	N2—N1—C1—N5	−179.0 (2)
N1^i^—Ni1—N1—N2	−33.46 (12)	Ni1—N1—C1—N5	14.9 (3)
O1^i^—Ni1—N1—N2	150.67 (13)	N2—N1—C1—N3	−0.1 (2)
O1^ii^—Ni1—N1—N2	−120.79 (13)	Ni1—N1—C1—N3	−166.30 (14)
C1—N1—N2—C2	0.6 (2)	C2—N3—C1—N1	−0.4 (2)
Ni1—N1—N2—C2	168.37 (13)	C2—N3—C1—N5	178.5 (2)
C1—N1—N2—Ni2	168.09 (13)	N1—N2—C2—N4	176.0 (2)
Ni1—N1—N2—Ni2	−24.17 (18)	Ni2—N2—C2—N4	9.6 (3)
N2^iv^—Ni2—N2—C2	−48.86 (15)	N1—N2—C2—N3	−0.9 (2)
N2^v^—Ni2—N2—C2	41.98 (14)	Ni2—N2—C2—N3	−167.25 (14)
N2^ii^—Ni2—N2—C2	132.67 (18)	C1—N3—C2—N2	0.8 (2)
N2^i^—Ni2—N2—C2	−136.44 (18)	C1—N3—C2—N4	−176.2 (2)

Symmetry codes: (i) −*y*+1, *x*−*y*, *z*; (ii) −*x*+*y*+1, −*x*+1, *z*; (iii) *y*+1/3, *x*−1/3, −*z*+1/6; (iv) *x*−*y*+1/3, −*y*+2/3, −*z*+1/6; (v) −*x*+4/3, −*x*+*y*+2/3, −*z*+1/6; (vi) −*y*+1, *x*−*y*−1, *z*; (vii) −*x*+*y*+2, −*x*+1, *z*; (viii) −*x*+2/3, −*y*−2/3, −*z*+1/3; (ix) *x*−*y*−1/3, *x*−2/3, −*z*+1/3; (x) *y*+2/3, −*x*+*y*+1/3, −*z*+1/3; (xi) −*y*, *x*−*y*−1, *z*; (xii) −*x*+*y*+1, −*x*, *z*.

### Hydrogen-bond geometry (Å, °)

**Table:**

*D*—H···*A*	*D*—H	H···*A*	*D*···*A*	*D*—H···*A*
O1—H11···O2	0.86 (3)	1.88 (3)	2.724 (3)	168 (3)
O1—H12···O3	0.85 (3)	1.89 (3)	2.737 (2)	174 (3)
O2—H21···O4	0.84 (3)	2.07 (3)	2.896 (4)	168 (4)
O2—H22···O3^xiii^	0.84 (3)	2.12 (3)	2.961 (3)	175 (4)
O3—H31···O4^ix^	0.85 (3)	2.05 (3)	2.861 (3)	159 (3)
O3—H32···F1^ix^	0.84 (3)	2.13 (3)	2.904 (2)	153 (3)
O4—H41···F2^xiv^	0.85 (3)	2.23 (3)	2.950 (3)	143 (4)
O4—H42···F3^xi^	0.84 (3)	2.16 (3)	2.973 (4)	162 (4)
O4—H41···O5	0.85 (3)	2.03 (3)	2.64 (2)	128 (4)
O5—H51···F1	0.85	1.92	2.76 (3)	167
N3—H3···F2^iii^	0.85 (3)	1.97 (3)	2.764 (2)	157 (3)
N4—H4b···F1^iii^	0.84 (3)	2.14 (3)	2.974 (3)	170 (3)
N5—H5a···O1^i^	0.84 (3)	2.23 (3)	2.942 (3)	142 (3)
N5—H5b···F3^xv^	0.85 (3)	2.08 (3)	2.909 (3)	167 (3)

Symmetry codes: (xiii) −*x*+5/3, −*y*+1/3, −*z*+1/3; (ix) *x*−*y*−1/3, *x*−2/3, −*z*+1/3; (xiv) *y*+1/3, *x*−4/3, −*z*+1/6; (xi) −*y*, *x*−*y*−1, *z*; (iii) *y*+1/3, *x*−1/3, −*z*+1/6; (i) −*y*+1, *x*−*y*, *z*; (xv) *x*, *y*+1, *z*.
